# 1052. Characteristics and Outcomes in Hospitalized Adult Patients Infected with the SARS-CoV-2 Omicron Variant at a Large Healthcare System in South Florida, January 1–14, 2022

**DOI:** 10.1093/ofid/ofac492.893

**Published:** 2022-12-15

**Authors:** Paula A Eckardt, Myeongji Kim, Ayesha T Jalal, Jessica E Goldberg, Elsa M Acevedo Martinez, Nathalie P Suarez Moscoso, Heysu Rubio-Gomez, Daniel Mayer, Alvaro Visbal, Candice Sareli, Jianli Niu, Aharon E Sareli

**Affiliations:** Memorial Healthcare System, Hollywood, Florida; Memorial Healthcare System, Hollywood, Florida; Memorial Healthcare System, Hollywood, Florida; Memorial Healthcare System, Hollywood, Florida; Memorial Healthcare System, Hollywood, Florida; Memorial Healthcare System, Hollywood, Florida; Memorial Healthcare System, Hollywood, Florida; Memorial Healthcare System, Hollywood, Florida; Memorial Healthcare System, Hollywood, Florida; Memorial Healthcare System, Hollywood, Florida; Memorial Healthcare System, Hollywood, Florida; Memorial Healthcare System, Hollywood, Florida

## Abstract

**Background:**

The SARS-CoV-2 Omicron variant has been rapidly spreading worldwide. We aimed to characterize Omicron severity by assessing in-hospital deaths and intensive care admissions in a large healthcare system in South Florida during an Omicron predominant surge.

**Methods:**

Laboratory-confirmed COVID-19 adult patients hospitalized during January 1—14, 2022 were retrospectively reviewed. Risks of in-hospital mortality and intensive care admission were estimated using logistic regression models. Analyses were stratified by age ≥ 65 years and vaccination status, and further adjusted for sex, comorbidities, and history of a previous COVID-19 infection.

**Results:**

500 consecutively hospitalized COVID-19 Omicron patients were included. The median age was 69 (IQR, 53-80) years, and 271 (54.2%) were women. The most common comorbidities were hypertension (65.5%), diabetes (32%), and chronic kidney disease (24%). 260 (52%) patients were fully vaccinated (defined as a patient who received 2-dose vaccines), and 32 (6.4%) were previously infected with COVID-19. 252 (50.4%) patients required supplemental oxygen, 54 (10.8%) required intensive care unit (ICU) admission, and 44 (8.8%) patients required mechanical ventilation. At study closeout of March 7, 2022, case fatality rates among patients aged 18–29 years, 30–39 years, 40-49 years, 50-59 years, 60-69 years, 70-79 years, and ≥ 80 years were 0%, 2.2%, 6.4%, 5.3%, 8.0%, 5.7%, and 15.4% respectively (*p*< 0.001), with the median time from hospital admission to death being 13 days (IQR, 6.5-20.5) (Figure 1). Patients aged ≥ 65 years had 2.6 times higher rates for in-hospital mortality (OR, 2.63; 95% CI, 1.29-5.33; *p*=0.007) than those aged < 65 years, but were comparable for ICU admission (OR, 0.85; 95% CI, 0.49-1.52; *p*=0.586). Past vaccination offered no protection against in-hospital mortality (OR, 1.18; 95% CI, 0.64-2.19; *p*=0.599) or ICU admission (OR, 1.16; 95% CI, 0.66-2.06; *p*=0.6) (Figure 2). In multivariable-adjusted models, patients aged ≥ 65 years had a higher in-hospital mortality than those aged < 65 years (Figure 2).

**Figure 1.**
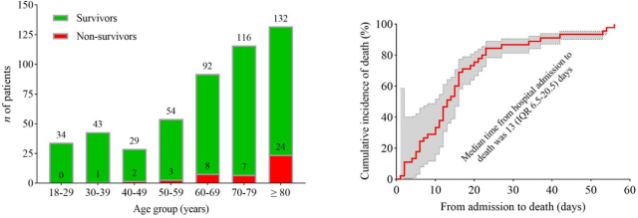
Left: Distributions of survivors and non-survivors among hospitalized COVID-19 Omicron patients at different age groups; Right: Death curve of non-survivors with COVID-19 omicron in the South Florida area, January 1-14, 2022.

**Figure 2.**
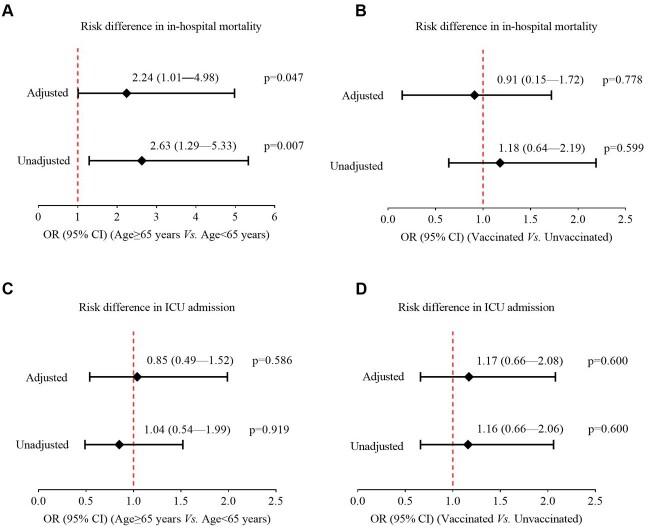
Forest plots showing association of age and vaccination status with COVID-19 Omicron patient outcomes. Results were reported as odds ratios (OR,•) with 95% confidence intervals (CIs, horizontal lines), adjusted for sex-, diabetes, hypertension, COPD, chronic kidney disease, coronary heart disease, active malignancy, history of malignancy, history of solid organ transplantation, history of bone marrow transplantation, HIV, and previous SARS-CoV-2 infection. A-B. In-hospital mortality (death); C-D. ICU admission. ICU, intensive care unit.

**Conclusion:**

This case series provides characteristics and outcomes of hospitalized adult patients with COVID-19 Omicron variant. Past COVID-19 vaccination did not impact ICU admission rate nor in-hospital mortality.

**Disclosures:**

**All Authors**: No reported disclosures.

